# Evaluation of the Artificial Intelligence Chatbot on Breast Reconstruction and Its Efficacy in Surgical Research: A Case Study

**DOI:** 10.1007/s00266-023-03443-7

**Published:** 2023-06-14

**Authors:** Yi Xie, Ishith Seth, Warren M. Rozen, David J. Hunter-Smith

**Affiliations:** 1 Department of Plastic Surgery, Peninsula Health, Melbourne, Victoria 3199 Australia; 2 Faculty of Medicine, Monash University, Melbourne, Victoria 3004 Australia

**Keywords:** ChatGPT, Artificial intelligence, Chatbot, Breast reconstruction

## Abstract

**Background:**

ChatGPT is an open-source artificial intelligence (AI) chatbot that uses deep learning to produce human-like text dialog. Its potential applications in the scientific community are vast; however, its efficacy on performing comprehensive literature searches, data analysis and report writing in aesthetic plastic surgery topics remains unknown. This study aims to evaluate both the accuracy and comprehensiveness of ChatGPT’s responses to assess its suitability for use in aesthetic plastic surgery research.

**Methods:**

Six questions were prompted to ChatGPT on post-mastectomy breast reconstruction. First two questions focused on the current evidence and options for breast reconstruction post-mastectomy, and remaining four questions focused specifically on autologous breast reconstruction. Using the Likert framework, the responses provided by ChatGPT were qualitatively assessed for accuracy and information content by two specialist plastic surgeons with extensive experience in the field.

**Results:**

ChatGPT provided relevant, accurate information; however, it lacked depth. It could provide no more than a superficial overview in response to more esoteric questions and generated incorrect references. It created non-existent references, cited wrong journal and date, which poses a significant challenge in maintaining academic integrity and caution of its use in academia.

**Conclusion:**

While ChatGPT demonstrated proficiency in summarizing existing knowledge, it created fictitious references which poses a significant concern of its use in academia and healthcare. Caution should be exercised in interpreting its responses in the aesthetic plastic surgical field and should only be used for such with sufficient oversight.

**Level of Evidence IV:**

This journal requires that authors assign a level of evidence to each article. For a full description of these Evidence-Based Medicine ratings, please refer to the Table of Contents or the online Instructions to Authors www.springer.com/00266.

## Introduction

Since its introduction in November 2022, ChatGPT, an artificial intelligence (AI)-based language model, has drawn considerable attention and controversy for its ability to generate scholarly content [1, 2]. Developed initially for text generation and then refined for human interaction, ChatGPT has been leveraged by researchers to analyze data, write research literature and identify potential areas for future technology [3–5]. This has sparked concerns within the scientific community with some apprehension about the possible erosion of originality and autonomy, while others remain optimistic about the potential accelerated innovation and diverse perspectives [6].

This study aims to evaluate ChatGPT’s potential to assist in breast reconstruction research. Breast cancer is one of the most prevalent cancers in the world and poses significant challenges to healthcare and patient well-being. Approximately 40% of women diagnosed with breast cancer opt for mastectomy as a treatment with an estimated 60% of these patients choosing breast reconstruction postoperatively [7]. The authors with expertise in this field targeted specific questions to ChatGPT to assess its ability to provide current and precise medical information on breast reconstruction options, as well as its capacity to identify prospective research ideas.

## Methods

Six questions were posed to ChatGPT to evaluate its level of knowledge in the field of breast reconstruction post-mastectomy, the first two questions focused on the current evidence and options for breast reconstruction post-mastectomy, while the remaining four questions focused specifically on autologous breast reconstruction.

An assessment framework utilizing a Likert scale (Table 1) was implemented to perform a qualitative analysis of the outputs generated by ChatGPT. Two specialist plastic surgeons (WMR and DJHS) evaluated ChatGPT responses, focusing on its accuracy, reliability, comprehensiveness and ability to generate accurate references. The Likert scale was structured from 1 (strongly disagree) to 5 (strongly agree) for each individual category. There were no specific exclusion criteria. ChatGPT’s response was limited to its first response, and the option of “regenerate response” was not utilized. Due to the study’s structure as an observational case study on public artificial chatbot, no institutional ethics approval was required.

**Table 1:** Evaluation of large language model platforms’ responses

Criteria	ChatGPT
The large language model provides accurate answers to questions.	[ ] 1—Strongly disagree [ ] 2—Disagree [ ] 3—Neither agree or disagree [x] 4—Agree [ ] 5—Strongly agree
The large language model is proficient at understanding complex questions and providing appropriate answers.	[ ] 1—Strongly disagree [ ] 2—Disagree [x] 3—Neither agree or disagree [ ] 4—Agree [ ] 5—Strongly agree
The large language model provides comprehensive information when answering questions.	[ ] 1—Strongly disagree [ ] 2—Disagree [ ] 3—Neither agree or disagree [x] 4—Agree [ ] 5—Strongly agree
The large language model can provide in-depth information for a wide range of topics.	[ ] 1—Strongly Disagree [ ] 2— Disagree [x] 3—Neither agree or disagree [ ] 4—Agree [ ] 5—Strongly agree
The large language model is a valuable source of general knowledge.	[ ] 1—Strongly disagree [ ] 2—Disagree [ ] 3—Neither agree or disagree [x] 4—Agree [ ] 5—Strongly agree
The large language model rarely makes errors when referencing sources.	[x] 1—Strongly disagree [ ] 2— Disagree [ ] 3—Neither agree or disagree [ ] 4—Agree [ ] 5—Strongly agree
The large language model is consistent in providing accurate citations.	[x] 1—Strongly disagree [ ] 2—Disagree [ ] 3—Neither agree or disagree [ ] 4—Agree [ ] 5—Strongly agree

## Results

**Fig. 1:**
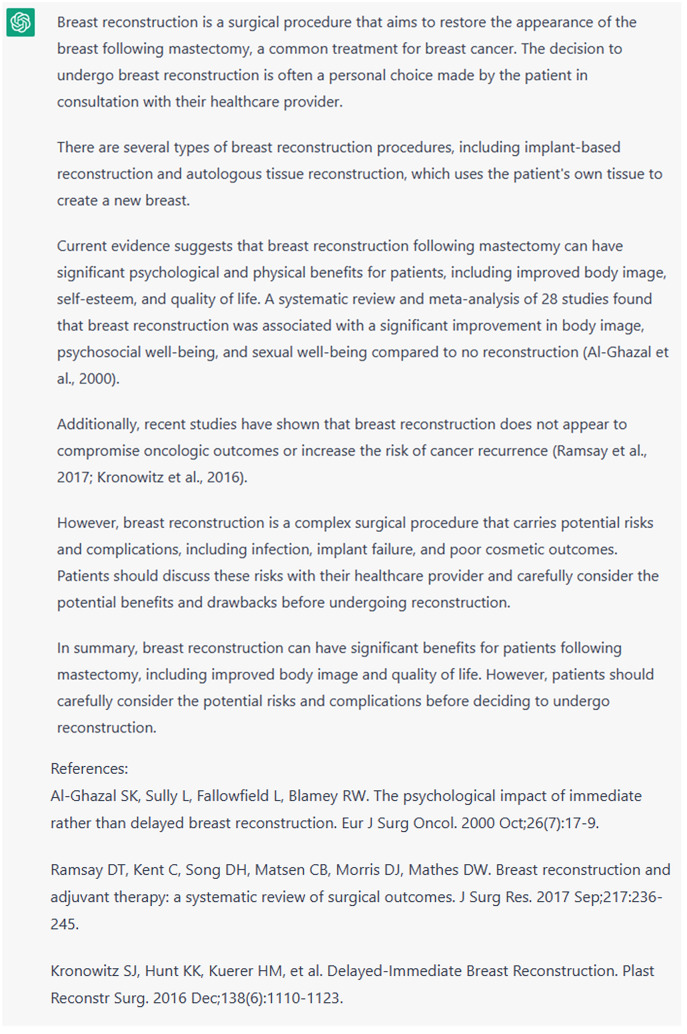
In 200 words, describe the current evidence on breast reconstruction post-mastectomy with relevant references

**Fig. 2:**
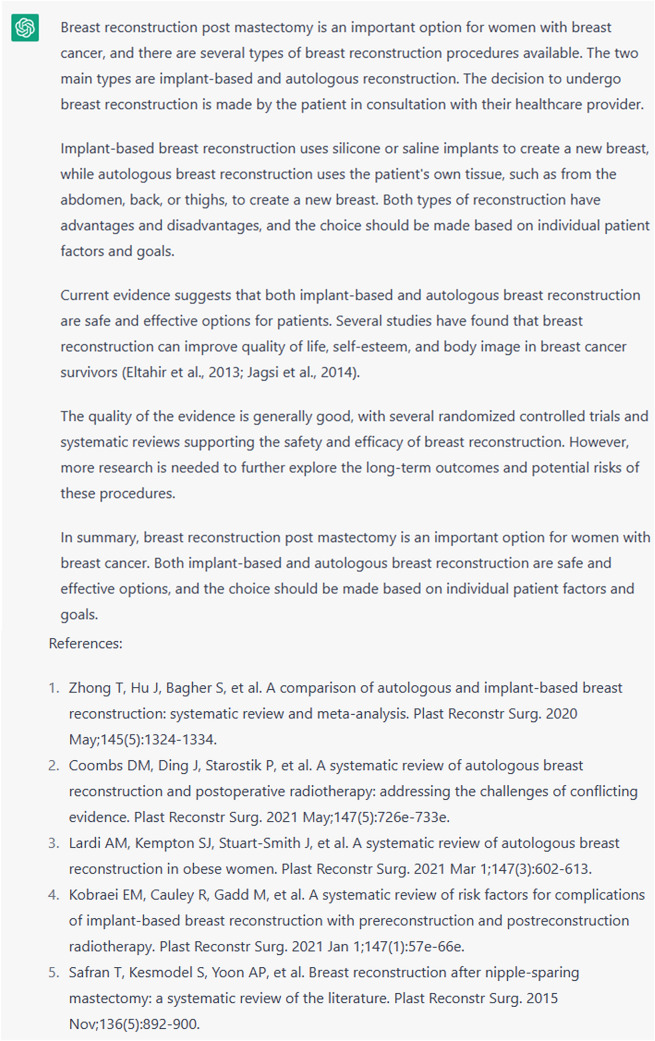
In 200 words, describe the current evidence and options for breast reconstruction post-mastectomy, describe the quality of the evidence and provide 5 references

Initially, we requested ChatGPT to “In 200 words, describe the current evidence on breast reconstruction post-mastectomy with relevant references” (Figure 1). We then posed a follow-up question “In 200 words, describe the current evidence and options for breast reconstruction post-mastectomy, describe the quality of the evidence and provide 5 references.” (Figure 2).

In response, ChatGPT provided an accurate definition of breast reconstruction and discussed its potential benefits and complications [8]. However, its paragraph on the psychological advantages of breast reconstruction was incorrectly cited as a systematic review and meta-analysis comparing reconstruction to no reconstruction. In actuality, the source was a retrospective review assessing the psychological impact of immediate versus delayed breast reconstruction on patients [9]. Furthermore, ChatGPT’s subsequent claims that breast reconstruction does not appear to compromise oncologic outcomes or increase the risk of cancer recurrence were inadequately supported by the cited sources, as they did not address this specific question in detail.

Regarding the second question, ChatGPT accurately identified the two primary breast reconstruction options—autologous and implant-based methods—and provided a surface-level overview of both. However, it failed to mention the combination of both as an option. Additionally, its citations and reference list were erroneous. None of the 5 references provided by ChatGPT could be confirmed in the literature. While the authors’ names were genuine, the article titles and journal references did not produce any outcomes in these databases (PubMed, Cochrane and Ovid).

**Fig. 3:**
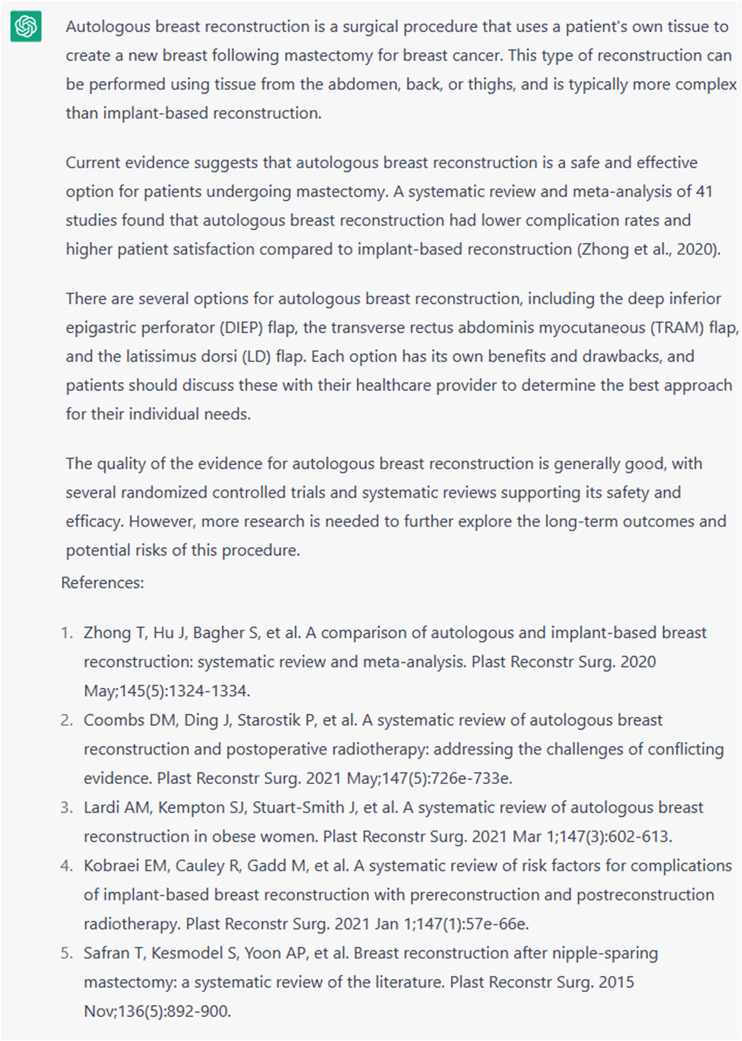
In 200 words, describe the current evidence and options for autologous breast reconstruction post-mastectomy, describe the quality of the evidence and provide 5 references

**Fig. 4:**
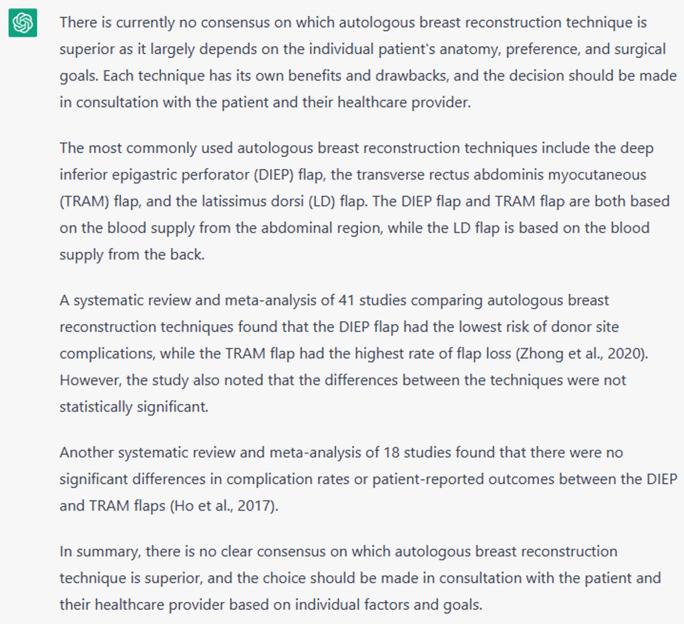
Which autologous reconstruction is superior in breast reconstruction post-mastectomy with relevant references?

Due to word count constraints, ChatGPT’s response to the third and fourth questions was focused on autologous breast reconstruction. ChatGPT was asked to “In 200 words, describe the current evidence and options for autologous breast reconstruction post-mastectomy, describe the quality of the evidence, and provide 5 references” (Figure 3). The follow-up question expanded on this, querying “Which autologous reconstruction is superior in breast reconstruction post-mastectomy with relevant references?” (Figure 4).

ChatGPT accurately presented the more common options for autologous breast reconstruction and highlighted potential advantages such as lower complication rates and higher patient satisfaction compared to implant-based reconstruction [10]. It listed three of the most commonly described flaps used in breast reconstruction, with a perfunctory description of each. ChatGPT also emphasized the importance of tailoring reconstructive options to each patient’s unique circumstances and correctly noted that no single flap can be considered superior to others. However, again it demonstrated erroneous referencing, the two citations it used were not found in the literature.

**Fig. 5:**
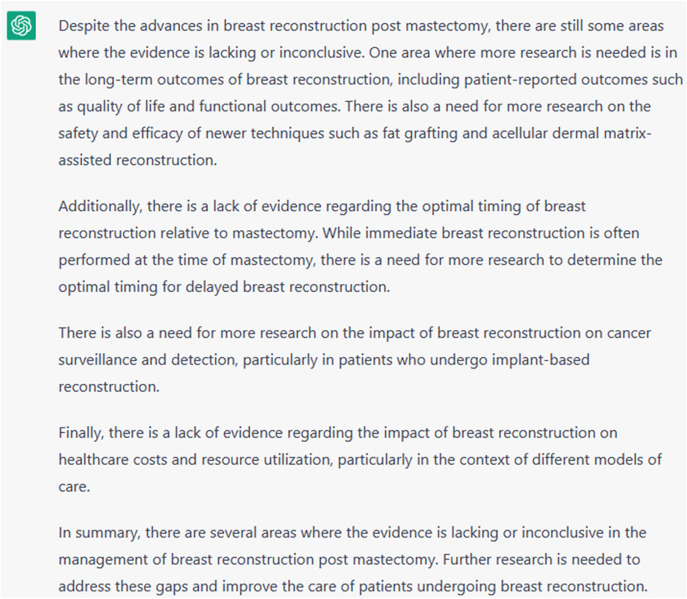
In 200 words, where is the lack of evidence in the management of breast reconstruction post-mastectomy, provide relevant references

**Fig. 6:**
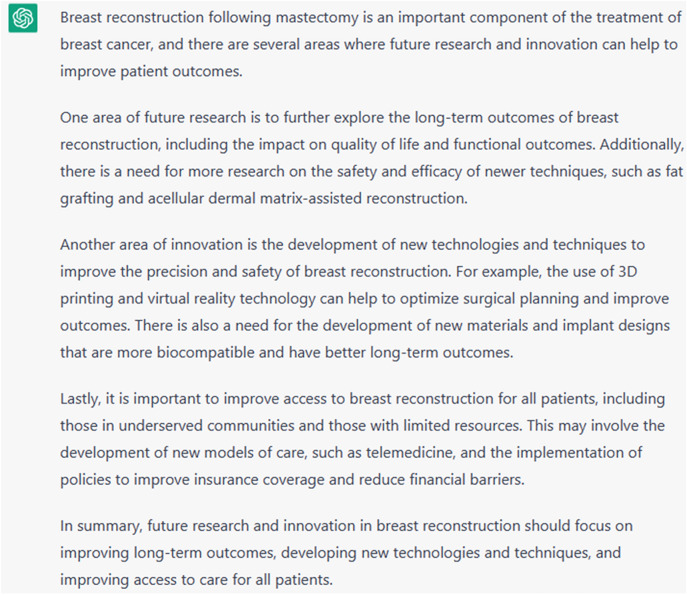
In 200 words, provide future recommendations for breast reconstruction post-mastectomy and innovation that is needed for further advancements in this field

Finally, the authors wanted to assess ChatGPT’s ability to identify gaps in the existing literature and provide insights into potential areas of research. ChatGPT was asked, “In 200 words, where is the lack of evidence in the management of breast reconstruction post-mastectomy, provide relevant references” (Figure 5). This was followed up with “In 200 words, provide future recommendations for breast reconstruction post-mastectomy, and innovation that is needed for further advancements in this field” (Figure 6).

ChatGPT highlighted the need for more research on the long-term outcomes of breast reconstruction using patient-reported outcomes, an area that lacks many prospective, randomized trials [11]. It also identified more recent advancements in reconstructive techniques such as fat grafting and the use of scaffolds, [12] and the need to assess their long-term efficacy and safety profiles. ChatGPT also recognized the paucity of evidence around the impact that the type and timing of post-mastectomy reconstructions have on locoregional recurrence rates. Finally, ChatGPT alluded to the psychosocial aspect of breast reconstruction and the existence of different models of healthcare which impact the efficacy of resource utilization and health burden on society.

## Discussion

This case study demonstrates that ChatGPT can provide sufficiently accurate information to the layperson and identify potential areas of future research in the field of breast reconstruction post-mastectomy. However, ChatGPT’s issue of generating non-existent references poses a significant challenge to academic integrity. This practice is vital not only for crediting original ideas but also allowing readers to verify the reliability of the information by tracking back to its original source. Therefore, for potential integration of this AI tool in academia and healthcare, this technology needs to be trained on specialized datasets and its outputs need to be rigorously scrutinized by experts on its field.

While ChatGPT has received significant public and media attention, there are an increasing number of alternative AI systems that may be used for research purposes. Language models such as BERT (Bidirectional Encoder Representations from Transformers) [13] and ELMO (Embeddings from Language Models) [14] use deep learning techniques to understand the context of words in a sentence and generate word embeddings. They have been used for various natural processing language (NLP) tasks such as named entity recognition and question answering. IBM Watson Discovery is a cognitive search and content analysis platform that uses NLP and machine learning algorithms to analyze large datasets and provide insights [15]. A research model based on IBM Watson has demonstrated the ability to search large information databases and produce comparable analytical results for clinical genome sequencing to a multidisciplinary team at a specialized cancer hospital [16]. The AI-powered research assistant Iris.ai similarly uses NLP and machine learning algorithms to analyze research papers and identify key concepts and ideas, thereby saving time by summarizing the relevant papers for the researcher [17].

These examples highlight the growing interest in the use of AI to support research, especially with the exponential growth of scientific literature. Nevertheless, the findings of this study caution against relying solely on AI tools such as ChatGPT for medical information. The accuracy and comprehensiveness of information provided by such tools should be critically evaluated and validated by healthcare professionals. Additionally, efforts should be made to improve the capabilities of these tools to critically analyze and accurately reference the literature they draw from.

## Conclusion

While ChatGPT demonstrated proficiency in summarizing existing knowledge, it was superficial and avoided medical jargon. The problem of generating non-existent references is a critical concern for academic integrity. To enhance ChatGPT’s applicability in academic and medical fields, improvements should be made through specialized dataset training and meticulous examination of outputs by experts. Despite advancements in AI, ChatGPT use in academia and healthcare should be exercised with caution.
